# Supplementary material for: Predictability of Radiologically Measured Psoas Muscle Area for Intraoperative Hypotension in Older Adult Patients Undergoing Femur Fracture Surgery
Source: J Clin Med. 2023 Feb 20;12(4):1691. doi: 10.3390/jcm12041691 (PMC9959025; doi:10.3390/jcm12041691)
Supplement: Supplementary file 1 [file jcm-12-01691-s001.zip › jcm-2173069-supplementary.pdf]

Table S1. Factors of modified frailty index

History of diabetes mellitus[a]
History of congestive heart failure
History of hypertension requiring medication
History of either transient ischemic attack or cerebrovascular accident
Non-independent functional status (partially or totally dependent activities of daily living)
History of myocardial infarction within 6 months
History of either peripheral vascular disease or rest pain
History of a cerebrovascular accident with neurological deficit
History of either COPD exacerbation or pneumonia within 30 days
History of either prior PCI, PCS at any time, or angina within 30 days
History of impaired sensorium

COPD, chronic obstructive pulmonary disease; PCI, percutaneous coronary intervention; PCS, prior cardiac surgery. [a] diabetes controlled by diet alone, treated with oral anti-hyperglycemic therapy or with insulin.
